# Mitochondrial genome of *Chalcidoidea* sp. (Hymenoptera: Apocrita: Chalcidoidea) and phylogenetic analysis

**DOI:** 10.1080/23802359.2020.1768954

**Published:** 2020-05-28

**Authors:** Yuanchen Zhang, Shuang Xue, Yilin Fan, Jingshun Wang, Kunpeng Zhang

**Affiliations:** a College of Biology and Food Engineering, Anyang Institute of Technology, Anyang, Henan, China; b College of Biology and Food Engineering, Innovation and Practice Base for Postdoctors, Anyang Institute of Technology, Henan, China; c Luoyang Entry-Exit Inspection and Quarantine Bureau of P. R. China, Luoyang, Henan, China

**Keywords:** Hymenoptera, mitochondrial genome, *Chalcidoidea* sp., phylogenetic analysis

## Abstract

Chalcidoidea (Hymenoptera) are minute wasps which can attack immature and adult stages of virtually all insect orders. Here, we sequenced and annotated the mitochondrial genome (mitogenome) of *Chalcidoidea* sp. This mitogenome was 15,152 bp long and encoded 13 protein-coding genes (PCGs), 20 transfer RNA genes (tRNAs), and 2 ribosomal RNA unit genes (rRNAs). All 13 PCGs were initiated by the ATN (ATG, ATT, ATA, and ATC) codon. All PCGs terminate with the stop codons TAA or TAG except for *nad4* which ended with the incomplete codon T−. Phylogenetic analysis showed that *Chalcidoidea* sp. got together with the species *Encyrtus infelix* and *Eurytoma* sp., and species in *Chalcidoidea* formed a sister group to other *Cynipoidea* and *Proctotrupoidea* species.

The superfamily Chalcidoidea is one of the most diverse groups of parasitic Hymenoptera. Most chalcid wasps are parasitoids that are successfully used as biological control agents of agricultural and ornamental pests, and they have tremendous importance in both natural and managed ecosystems (Gibson et al. 1999; Munro et al. 2011).

Specimens of *Chalcidoidea* sp. were collected from Baoji City, Shaanxi Province, China (33°51′N, 107°22′E, June 2019) and were stored in Entomological Museum of Anyang Institute of Technology (Accession number AIT-E-CHA06). After morphological identification, total genomic DNA was extracted from tissues using DNeasy DNA Extraction kit (Qiagen, Hilden, Germany). The mitogenome sequence of *Chalcidoidea* sp. was generated using Illumina HiSeq 2500 Sequencing System. In total, 6.9 G raw reads were obtained, quality-trimmed, and assembled using MITObim v 1.7 (Hahn et al. 2013). By comparison with the homologous sequences of other Chalcidoidea species from GenBank, the mitogenome of *Chalcidoidea* sp. was annotated using software GENEIOUS R8 (Biomatters Ltd., Auckland, New Zealand).

The nearly complete mitogenome of *Chalcidoidea* sp. is 15,152 bp (Genbank accession, MT419887). It contains 13 protein-coding genes (PCGs), 20 tRNA genes, 2 rRNA genes, and a partial non-coding AT-rich region. Mitogenome of *Chalcidoidea* sp. exhibit dramatic mitochondrial gene rearrangement which is common in Chalcidoidea species (Zhu et al. 2018; Tang et al. 2019; Xiong et al. 2019). The overall base composition of the mitogenome was estimated to be A 46.5%, T 37.3%, C 10.1%, and G 6.1%, with a high A + T content of 83.8%. All 13 PCGs of *Chalcidoidea* sp. have the conventional ATN start codons for invertebrate mitochondrial PCGs (six ATT, five ATG, and one each of ATA and ATC). Most of the PCGs terminate with the stop codon TAA or TAG, whereas *nad4* end with the incomplete codon T. Two rRNA genes (*rrnL* and *rrnS*) located at *trnL1*/*trnA* and *trnA*/*trnV*, respectively. The lengths of *rrnL* and *rrnS* in *Chalcidoidea* sp. were 1301 and 776 bp, with the AT contents of 85.9 and 86.2%, respectively. Two tRNA genes (*trnC* and *trnY*) were not predicted in this mitogenome, and the other 20 tRNA genes vary from 64 bp (*trnI* and *trnS1*) to 74 bp (*trnQ*).

All 13 mitochondrial protein-coding genes sequences were extracted from the mitochondrial DNA sequences of 17 related taxa of Apocrita. A phylogenetic tree was constructed through raxmlGUI 1.5 (Silvestro and Michalak 2012). Results showed that the newly sequenced species *Chalcidoidea* sp. got together with the species *Encyrtus infelix* and *Eurytoma* sp., and all 11 Chalcidoidea species constituted one clade and formed a sister group to other Cynipoidea and Proctotrupoidea species. (Figure 1). In conclusion, the mitogenome of *Chalcidoidea* sp. is sequenced in this study and can provide essential DNA molecular data for further phylogenetic and evolutionary analysis of Apocrita.

**Figure 1.**
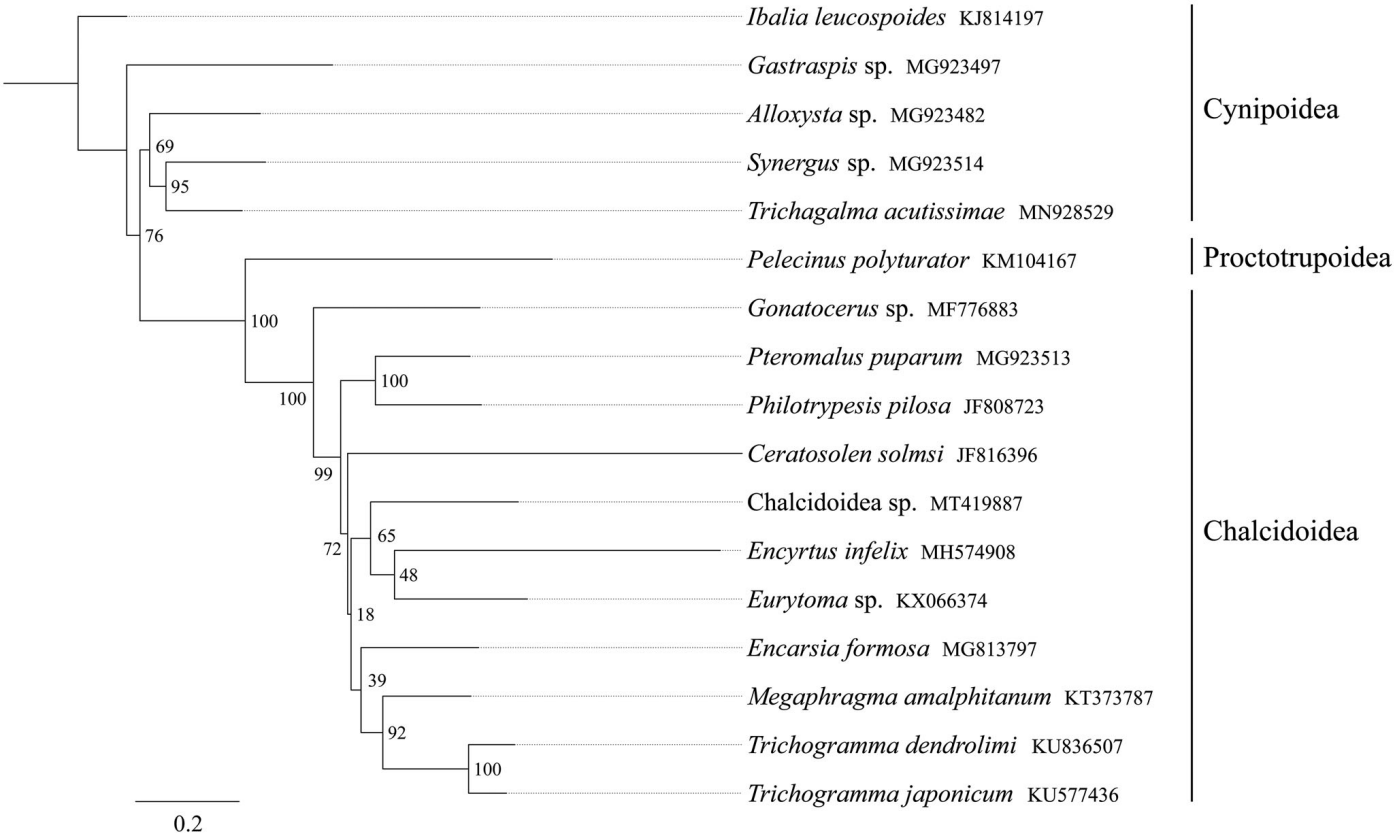
Phylogenetic relationships based on the 13 mitochondrial protein-coding genes sequences inferred from RaxML. Numbers on branches are Bootstrap support values (BS).

## Data Availability

The data that support the findings of this study are openly available in NCBI (National Center for Biotechnology Information) at https://www.ncbi.nlm.nih.gov/, reference number MT419887.
